# Pheochromocytoma and Paraganglioma: A Review of Diagnosis, Management and Treatment of Rare Causes of Hypertension

**DOI:** 10.7759/cureus.7969

**Published:** 2020-05-05

**Authors:** Ana Cerqueira, Tiago Seco, Ana Costa, Maria Tavares, Jorge Cotter

**Affiliations:** 1 Internal Medicine, Hospital Senhora Da Oliveira, Guimarães, PRT

**Keywords:** hypertension, paragangliomas, pheochromocytomas

## Abstract

Pheochromocytomas (PHEO) and paragangliomas (PGL) are rare tumors originated in cells derived from the neural crest. The first ones are located in the adrenal medulla, and the second ones in the sympathetic and parasympathetic nervous system. These kind of tumors may secrete excess catecholamines, including epinephrine, norepinephrine, dopamine and/or their metabolite metanephrine, normetanephrine and 3-methoxytyramine, respectively. Its clinical manifestations depend on the location, the secretory profile and the malignant potential of the tumor. These tumors are frequently benign in their presentation. Some arise in the context of familiar syndromes, accounting for up to one-third of the total of diagnosis. The metastatic form is the most common presentation of the tumors with familiar origin and due to their rarity, their diagnosis and management is often difficult. Over the years, our knowledge and perception of PHEO and PGL has greatly expanded and changed. This review article aims to focus on the genetic, clinical, diagnostic, therapeutic and prognostic approaches, to give the clinician knowledge of the most recent updates regarding these themes.

## Introduction and background

Pheochromocytomas (PHEO) and paragangliomas (PGL) are rare neuroendocrine tumors that produce catecholamines and originate from the adrenal medulla chromaffin cells (80%) or from neural crest cells outside the adrenal gland (20%) [1]. According to the World Health Organization (WHO), PHEO concern tumors located in the adrenal gland and the PGL are tumors of extra-adrenal localization. PGL can still be divided into two subtypes: parasympathetic or sympathetic [2]. This last kind is typically originated in the chromaffin cells of the sympathetic paraganglionic chains of the chest and abdomen and are catecholamine-producing tumors. The annual incidence of PHEO is located between 500 and 1,600 cases per year, with an equal sex distribution and a peak in the fourth and fifth decades of life [3]. The prevalence of PHEO in patients with hypertension is 0.1%-0.6%. The classical clinical features of PHEO consist of episodic hypertension, headaches, diaphoresis and flushing, but these features only occur in about 40% of the cases [4]. PGL affects approximately 0.1% of individuals with hypertension [5]. Most PGL are benign; however, a few of them are diagnosed with metastasis and in this case patients' five-year survival rate is between 40% and 77%, with a progression-free survival period that ranges from 4 to 36 months [6]. Metastatic PGL behave in a variable manner, with some initially presenting with metastases and some developing metastases several years after the initial diagnosis. It is now well established that there are several factors correlated with an accelerated disease progression, including male sex, diagnosis at an older age, synchronous metastases, bigger tumor size, increased dopamine level and failure to remove the primary tumor [5]. PHEO and PGL can be characterized into localized or multifocal. In the case of PHEO, they can be unilateral or bilateral and in the case of PGL it can be present in one or more paraganglionic chains. Both PHEO and PGL may be associated with metastatic disease, and this happens when we find chromafim tissue in places where it is not expected (usually in bone, lung, liver and lymph nodes histologically confirmed) [3]. The etiology of these tumors can be sporadic or familial. According to the latest WHO 2017 report, it is estimated that about 30% of these tumors originate in the context of hereditary disease. Currently, there are approximately 19 germline mutations in genes of increased susceptibility to PHEO/PGL. These commonly appear in the context of hereditary disease, but somatic mutations in the same genes have also been identified in the context of sporadic disease [5]. Germline genetic mutations linked to the succinate dehydrogenase (SDH) enzyme complex are the most frequent and are associated with familial syndromes [2]. Other germline mutations, which often predispose to the emergence of PHEO and PGL, include mutations in the rearranged during transfection (RET) proto-oncogene (associated with multiple neoplasia type 2 endocrine [MEN2]), mutations in the tumor suppressor gene Von Hippel-Lindau [VHL]) and mutations in the neurofibrin gene (neurofibromatosis type 1 [NF1]) [3]. The genotype-phenotype correlations determine the tumor location, hormonal function, multiplicity, risk of metastasis and associated syndromic symptoms [6]. A proportion of 11%-24% of the patients with PHEO/PGL with forms apparently sporadic (that is, without a positive family history or syndromic forms) have genetic mutations [3]. As an example, in the 2002 study by Neumann et al., which gathered 271 cases of PHEO and PGL associated with syndromic diseases, a proportion of 24% of mutations were identified in the germinal mutations [6]. This study, as well as subsequent ones, changed the paradigm regarding the heredity of this type of tumors, which was estimated to be present in only 10% of the cases (before 2000, the year in which SDH mutations began to be identified) [7].

## Review

PHEO and PGL are tumors that produce catecholamines (adrenaline, norepinephrine and dopamine). Often, production and catecholamine secretion are episodic and variable; with the excessive production and secretion of both noradrenaline (NA) and adrenaline, there is usually a predominance of NA. In less often cases, these can also be produced in isolation, and excess dopamine production rarely occurs [8]. For this reason, PHEO and PGL are tumors with an extremely variable clinical presentation and may present a myriad of signs and symptoms, ranging from a complete lack of symptoms to cases of sudden death [9]. They are characterized by the classic triad of features composed of headache, diaphoresis and tachycardia (with or without palpitations). However, this presentation is not mandatory, occurring in only 24% of the cases [10]. The concomitant presentation of these three symptoms associated with arterial hypertension has a diagnostic specificity greater than 90% [4]. Despite the low prevalence of induced hypertension by PHEO/PGL in the general population, hypertension is the most frequent cardiovascular manifestation, present in about 90% of patients with these tumors and usually it is persistent hypertension [5]. However, there are patients who, in addition to persistent hypertension, present paroxysmal tension peaks and still others who present only paroxysmal hypertension. In a study of 201 cases of PHEO and PGL done by Kopetschke et al., it was found that hypertension was present in 93.9% of the cases. Of these, about 50% had persistent hypertension, 36% had combined persistent and paroxysmal arterial hypertension and 8% had isolated paroxysmal hypertension [11]. There are also other clinical findings that may be present, namely orthostatic hypotension, pallor, chest pain, dyspnea, weight loss, heat intolerance, nausea, vomiting, constipation and psychiatric disorders (panic attacks and anxiety disorders). However, these are much less frequent [12]. All of these symptoms can occur in isolation, and given their nonspecific character, the diagnosis is sometimes a very difficult challenge. Occasionally, PHEO and PGL can cause paraneoplastic syndromes through the ectopic production of regulatory peptides, the most common being Cushing's syndrome, which results from the ectopic production of adrenocorticotropic hormone (ACTH) [1]. Between 5% and 15% of patients are normotensive and are bereft of any type of clinical manifestation [13]. In these cases, the diagnosis is based on the existence of family illness or clarification of the nature of adrenal incidentaloma [5]. Currently, around 30% of cases are diagnosed following investigation of imaging findings [8]. However, it should be noted that only 5% of the incidentalomas correspond to PHEO. In patients with hereditary disease, signs and symptoms may result from anomalies associated with syndromic forms, and usually their clinical presentation precedes the PGL or PHEO hypothesis. In such cases, an early diagnosis is possible.

Diagnosis is made through a combination of laboratory and imaging tests. PHEO and PGL are diagnosed through the analytical evidence of excessive production of catecholamines or their metabolites. Adrenaline and norepinephrine are metabolized by catecholamine-O-methyl transferase in metanephrine and normetanephrine, respectively (inactive metabolites). The production and secretion of catecholamines by the tumor is often reduced and episodic in character, unlike that of its inactive metabolites whose production is performed continuously, by a process that is independent of exocytic secretion of catecholamines. Consequently, metanephrine measurement in both plasma and urine is an excellent diagnostic method and is currently recommended as an initial method of diagnosis, according to the recommendations of clinical practice of the Society of Endocrinology (JCEM 2014) [13]. Recent studies indicate that analysis with the highest diagnostic sensitivity can be achieved through the measurement of free metanephrines in plasma, in which the sensitivity score lies in the range of 97%-99% [14]. Thus, a negative result is usually enough to exclude the diagnosis; however, some PGL produce only dopamine. In the study by Lenders et al., a comparative analysis of the diagnostic sensitivity of the measurement of metanephrines (both plasma and urine), vanillmandelic acid, and serum and urinary catecholamines was performed, and it was found that metanephrine measurement plasma concentrations produced significantly better results compared to other methods, whose sensitivities were 64%, 84% and 86%, respectively [2]. The biochemical profile can also be useful in identifying the type of tumor. Unlike PHEO, sympathetic PGL rarely secrete adrenaline, since the enzyme needed to convert norepinephrine to adrenaline (phenylethylamine N-methyltransferase) is expressed exclusively in the adrenal glands, consequently producing only norepinephrine and dopamine [15]. Moreover, the hereditary forms of these diseases have some differences. MEN2 and NF1 usually result in the production of adrenaline, whereas in the Von Hippel-Lindau syndrome, mainly NA is produced [16]. Imaging methods are used for diagnostic confirmation in order to locate and evaluate the tumor mass anatomically and functionally, allowing subsequent planning of the therapeutic approach. Both abdominal CT and MRI are useful as early imaging methods. However, CT is preferred given its high cost-effectiveness and sensitivity value, managing to detect PHEO at least 0.5 cm in diameter with a sensitivity of 85%-94% for PHEO and approximately 90% for PGL [12]. The MRI allows a better characterization of the tumor and the surrounding environment, enabling the exclusion of vascular invasion. Similarly, it allows a better distinction between soft tissues, and is thus superior in its ability to differentiate between PHEO and adrenal adenomas [12]. MRI diagnostic sensitivity lies between 93% and 100% for PHEO and approximately 90% in cases of PGL, metastasis or recurrence [4]. However, both MRI and CT have low specificity (approximately 70%-80%), increasing to about 100% when combined with 123I meta-iodobenzylguanidine scintigraphy (123I-MIBG) [5]. 123I-MIBG scintigraphy has an extremely high diagnostic specificity of 95%-100% and a sensitivity of 77%-90% [4]. It confirms, from the functional point of view, the location of the tumor tissue derived from CT or MRI. It is also useful in the diagnosis of extra-adrenal tumors and in the identification of tumor tissue after initial surgical removal, when tumor recurrence or excision is suspected to be incomplete. Positron emission tomography (PET) is also an alternative imaging test, with new agents such as 18F-deoxyglucose (F-FDG), 18F-dihydroxyphenaline (F-DOPA) and 18F-fluorodopamine (F-FDA) that can be used in cases of negative 123I-MIBG when there is a high clinical and laboratory suspicion. In particular, PET with 18F-FDG has a greater sensitivity than 123I-MIBG scintigraphy for metastatic disease, since in these cases, the tumors are generally less differentiated and consequently lose their ability to efficiently capture the I-MIBG [12]. The technique was therefore part of the recommendations of the Society of Endocrinology presented at JCEM in 2014. In addition, genetic study is extremely important in view of the high proportion of genetic mutations found in apparently sporadic forms of disease, and its performance is currently recommended in all patients with PHEO and PGL [13].

Tumor resection is the gold-standard treatment for disease related both to PHEO and PGL, and it is the only potentially curative therapeutic modality [12]. The laparoscopic approach is preferred because it is usually associated with shorter hospital stays and a lower rate of postoperative complications [13]. Open surgery is also a valid alternative, especially in cases of invasive disease to ensure complete tumor resection, prevent rupture and avoid local recurrence, constituting the preferred approach in cases of PGL, multifocal disease and tumor size greater than 6 cm [13]. In case of intra-adrenal sporadic and/or unilateral disease, total adrenalectomy is the gold-standard approach [10]. In the presence of bilateral PHEO, an adrenalectomy that saves adrenal cortex would be the ideal approach, since it avoids permanent hypocortisolism [17]. Similarly, in recurrent disease, if the contralateral adrenal has already been removed, the above approach also emerges as a therapeutic option [12]. In metastatic disease, treatment is also based on tumor resection surgery with a palliative aim of reducing tumor volume and consequent reduction of circulating catecholamines, given their direct relationship with mortality and patient morbidity [18]. In cases of unresectable disease and in those where the surgery is not curative, chemotherapy and/or radionucleotherapy can be used (with 131I-MIBG essentially) [19]. In a retrospective analysis of 116 cases of PHEO and malignant PGL treated with 131I-MIBG, a symptomatic improvement was reported in up to 75% of the cases: 45% obtained a biochemical response and 30% an imaging response. Patients who responded initially to therapy survived for an average of 22 months, about nine months more than non-responsive ones [20]. In the absence of an I-MIBG response or an unsatisfactory clinical response, other therapies may be used, namely octreotide (radiopharmaceutical therapy similar to that involving the use of somatostatin) or the chemotherapy, which includes cyclophosphamide, vincristine and dacarbazine (CVD) [21]. However, there is still a scarcity of controlled studies that can validate the effectiveness of these strategies in clinical practice, which is why their success has been limited to date.

In non-metastatic disease, regardless of whether it is PHEO or PGL, the five-year survival rate is higher than 95% [12]. Currently, it is admitted (as reflected in the fourth edition of WHO's classification 2017) that all PHEO and PGL have some metastatic potential [15]. The previous 2004 WHO classification of PHEO/PGL as “benign” or “malignant” at the time of diagnosis is of little use and should instead be replaced by the concept of risk stratification, with the implicit understanding that any PHEO or PGL has the potential to metastasize, sometimes many years after diagnosis [21]. Compared to PHEO, sympathetic PGL present a higher risk of metastasis [2]. In a meta-analysis of 10 cases, a 2.1 to 4.9 times higher risk of metastasis for PGL was reported compared to PHEO (a 10% risk of metastasis for PHEO in contrast to the average risk of 40% for sympathetic PGL) [22]. However, the risk of metastasis presents a close correlation with the genotype, which may vary between 2.5% and 50%, depending on the mutation found [12]. The mutation in the SDH subunit B gene is the one that poses the greatest risk of development of metastatic disease [16]. There are other risk factors for metastasis, including tumor mass greater than 5 cm, advanced age at the time of diagnosis and tumors with a high dopamine production [22]. Histological evaluation and immunohistochemical study are not truly predictive of tumor behavior in the long term; however, in retrospective studies, some common characteristics were identified between the cases of PHEO and PGL that metastasized [23]. There are five main parameters: invasion (vascular or peritumoral soft tissue), architectural variations (diffuse, irregular, confluent growth), cytological variations, necrosis and activity proliferation (atypical mitoses, proliferative index) identified by the grading system for adrenal PHEO and PGL whose result allows the assessment of the risk of metastasis and possibility of survival [24]. After the development of metastatic disease, the survival rate five years after diagnosis varies between 34% and 60%; however, there are many cases with an extremely aggressive evolution associated with a two- to four-year survival period, as well as cases of patients with indolent evolutions that survive 20 years or more after diagnosis [1,17]. In these cases, therapeutic approaches are, as mentioned above, limited and mostly palliative, so these patients always have a poor vital prognosis. Given the prognostic differences due to the onset of metastatic illness and the temporal unpredictability of its appearance, it is important to emphasize the central role of patients in terms of long-term follow-up. In 2014, the recommendations of clinical practice elaborated by JCEM came to suggest follow-up with all patients with PHEO or PGL, regardless of the risk of recurrence initially estimated [13]. This must be annual, with biochemical profile control in order to ascertain the presence of persistent, recurrent, or metastatic disease [12].

## Conclusions

In this review, we aim to raise awareness regarding the importance of a high rate of clinical suspicion for PHEO and PGL for a timely and effective diagnosis. We also intend to emphasize the importance of framing the clinical manifestations of the disease in the particular patient that we are evaluating because, not infrequently, the existence of comorbidities can skew or hinder the diagnosis which is already not easy under normal circumstances. The role of complementary means of diagnosis, namely in the early detection of these tumors in pre-symptomatic stages or in patients with more atypical manifestations, is equally important, because in about one-third of the cases, it is the clarification of incidentaloma that leads to the diagnosis of these entities. We want simultaneously to emphasize how new approaches in this field can promote greater diagnostic efficiency, particularly in cases of PGL and metastatic disease, playing a pivotal role in patient follow-up. Finally, we aim to highlight the prognostic implications. Given the metastatic potential of PGL and the temporal unpredictability of the appearance of metastases, the follow-up of these patients is essential and should be done for an unlimited period of time.
